# Male out-migration and the health of left-behind wives in India: The roles of remittances, household responsibilities, and autonomy

**DOI:** 10.1016/j.socscimed.2021.113982

**Published:** 2021-07

**Authors:** Lei Lei, Sonalde Desai

**Affiliations:** a Department of Sociology,Rutgers University, Davison Hall, 26 Nichol Avenue, New Brunswick, NJ, 08901, USA; b Department of Sociology, Maryland Population Research Center, University of Maryland, College Park 2112 Parren Mitchell Art-Sociology Building, 3834 Campus Dr, College Park, MD, 20742, USA

**Keywords:** Migration, Left-behind wives, Autonomy, Responsibilities, Remittances, Self-rated health

## Abstract

In developing countries, labor out-migration has led to millions of married couples living apart from each other. Male out-migration brings economic benefits to the families in places of origin, but also leads to profound changes in the lives of the left-behind wives. It is unclear how the husband's out-migration influences the health of wives, let alone the mechanisms through which any effects are transmitted. Using data from the Indian Human Development Survey (2004–2005 and 2011–2012), we estimated lagged dependent variable models (N = 19,737) to assess the health impact of husbands' out-migration for women in India. The results showed that left-behind wives had lower self-rated health than wives of non-migrants. Part of this negative health impact was driven by the low remittances sent by the migrant husbands. For both women in nuclear families and women in extended families, the negative health impact was partially attributable to women's added responsibilities, such as animal care and managing a bank account. For women in nuclear families, the negative health effect of husbands' migration has been partially suppressed by women's increased autonomy.

## Highlights

• Husbands' out-migration had a negative overall health impact on Indian women.
• Part of the negative health impact was driven by low remittances.
• Women's added responsibilities partially explained the negative influence.
• Women's autonomy partially suppressed the negative effect of husbands' migration.

The massive flow of internal and international migration of male workers geographically separates millions of women in developing countries from their husbands. Previous literature has emphasized the economic benefits that out-migration brings to the families in origin communities (De Brauw and Rozelle, 2008; Mberu, 2006; Taylor et al., 2003). However, male out-migration also leads to marital separation and family disruption, elevating stress among wives left behind. The absence of the husbands also brings profound changes in the lives of wives staying behind by increasing their workloads (Mu and Van de Walle, 2011) and family responsibilities on the one hand (Gulati, 1993), and granting more decision-making power and autonomy to the wives on the other hand (Desai and Banerji, 2008; Hadi, 2001). While the increased workloads and responsibilities can create physical strain, the improved economic conditions and enhanced autonomy could be conducive to the health of left-behind wives. However, few studies have considered the less immediate link between male out-migration and the general health of left-behind wives, let alone the mechanisms through which the health effect is transmitted.

A handful of studies on the impact of spousal migration on the health of left-behind wives have reported mixed findings. Some studies reported negative effects of husbands' absence on the mental health and reproductive health of left-behind wives (Lu, 2012; Roy and Nangia, 2005; Sevoyan and Agadjanian, 2010). However, they did not find that left-behind wives differ from wives of non-migrants in general morbidity or self-rated health (Chen et al., 2015; Roy and Nangia, 2005). A possible reason for the null findings could be that the positive and negative mechanisms through which male out-migration influences women's health tend to offset each other, resulting in a small and non-significant total effect on health. The current study seeks not only to assess the overall effect of husbands' out-migration on the health of left-behind wives but also to explore the indirect effects through several countervailing mechanisms. Moreover, we consider the distinct situations of wives living in nuclear families and those living with extended families, as the health consequences of husbands' out-migration could operate through different mechanisms for women with different living arrangments.

Relying on the rich nationally representative data from the Indian Human Development Survey (IHDS) collected in 2004-05 and 2011-12, we explore three research questions. First, does husbands' out-migration affect women's self-rated health? Second, how does husbands' out-migration influence economic conditions, household responsibilities, and autonomy of women in nuclear families and extended families? Third, do changes in these domains help explain the effects of husbands' migration on wives' health with different living arrangements?

India is an interesting setting to examine these questions because solo male migration is a common livelihood strategy adopted by families in India. Migrant men often leave their wives and children in the place of origin because of the low income, uncertain employment conditions, and expensive housing in migration destinations, and the need to care for land or older parents in the origins. In regions like Bihar and Uttar Pradesh, a tradition has emerged where men live in large cities for decades, visiting their families twice a year (Deshingkar et al., 2008; Gulati, 1993). Moreover, given the common practice of married couples living with the husbands' family in India, the study is able to explore the changes in family dynamics and the health consequences for women in extended and nuclear families after husbands’ migration.

## 1 Theory and literature

Deploying the theories linking marriage and health and literature on spousal migration and women's well-being in developing countries, we explain how husbands' out-migration can have both beneficial and detrimental effects on the health of left-behind wives in India. Previous research has recognized the emotional strain related to marital dissolution and its negative health implications (Williams and Umberson, 2004). Analogously, spatial separation between spouses can lead to marital instability and emotional pain. Qualitative studies have shown that left-behind women tend to feel lonely, miss their husbands, worry about their safety, and be upset about their husbands' potential relationships with other women in migration destinations (De Snyder, 1993; Menjívar and Agadjanian, 2007; Roy and Nangia, 2005). The long-term absence of the migrant spouse can reduce closeness and intimacy in the marital relationship (Menjívar and Agadjanian, 2007). Therefore, living separately from migrant husbands may raise stress levels of women staying behind and consequently undermine their health. In addition to this direct emotional strain, husbands' migration tends to influence women's health through changes in economic resources, women's responsibilities, and autonomy.

### 1.1 Economic resources as a mechanism

Women married to migrants benefit less from the economic and social resources provided by marriage than other women (Carr and Springer, 2010). After the husband migrates, the couple can no longer enjoy economies of scale due to geographic separation. The husbands' migration trips also require extra household spending to cover related expenses, increasing the economic strain of the origin households. After settling down, migrants usually send remittances to help improve the economic conditions of the origin households (Aghajanian et al., 2014; Hadi, 1999; Hugo, 2002). Remittances can be used to sustain food security, improve housing quality, and meet social obligations (Paris et al., 2005), potentially resulting in better health outcomes for women staying behind. In case the migrant males cannot secure jobs in the destinations, the small and irregular remittances may not compensate for the lack of the husband's labor or his previous salary. Staying-behind wives then face economic hardship, a chronic stressor that can alter the balance of the body's endocrine and immune system, leaving the body more susceptible to various disease agents (Cassel, 1976). Overexposure to stress hormones can result in allostatic load and negative health consequences, such as cardiovascular diseases, brain dysfunction, and increased susceptibility to communicable diseases (McEwen, 1998). Economic difficulties can also drive staying-behind wives to work more hours and reduce household expenditures (Paris et al., 2005), thus elevating the physical strains their bodies bear and reducing the necessary nutrition and care that they receive. Thus, part of the negative health implications of male migration could be attributable to the limited remittances sent by the migrants.

### 1.2 Women's responsibilities as a mechanism

Married partners living together can provide social support and share responsibilities in household management and family production (Carr and Springer, 2010). Out-migration disrupts such day-to-day instrumental support provided by the husbands. In a patriarchal society, men usually act as the household head and take responsibilities for managing household affairs, family business, and agricultural production. In the husbands' absence, the wives undertake more responsibilities in farming, family business, household management, and child-rearing (De Snyder, 1993; Mu and Van de Walle, 2011; Paris et al., 2005; Tong et al., 2019). For instance, in rice-producing villages of Uttar Pradesh, India, women undertook a broader range of farm tasks and a heavier workload to compensate for the absence of migrant husbands’ farm labor (Paris et al. 2005). Left-behind women in China also spent more hours in agricultural work than women in non-migrant households (Mu and Van de Walle, 2011).

In Indian society, the lack of instrumental support from the husband is especially challenging for women due to the practice of *purdah* or female seclusion (Desai et al., 2010). Under this gender restrictive norm, women are expected to be physically segregated from male strangers and senior male relatives and need to cover their bodies in public. The norm also confines women's activities to the domestic realm and prevents their intrusion into public space. However, left-behind wives have to go to public places to complete tasks previously carried out by their husbands, such as going to banks and post offices and interacting with various government institutions (Gulati, 1993). These unfamiliar and challenging tasks can add stress to women's daily lives. Because completing these tasks may violate the local gender norms, the judgments and gossips in the communities could make the tasks more burdensome for wives left behind.

Regarding the health impacts of the added roles and responsibilities, the prolonged work hours and the many behavioral readjustments required in a short period after men left could overtax women's abilities to cope and increase their stress, leaving them more susceptible to various infection, injury, or disease (Thoits, 2010). Venturing in the community without a male company also generates safety concerns for women, especially given the prevalent sexual harassment in some places. Therefore, we, in general, expect that male out-migration increases women's responsibilities in the household and on the farm, which then affects their health negatively.

In some situations, women may adjust well, learn from new experiences, and even gain more self-confidence. For example, exposure of women in migrant households to banks has broadened their vision on financial issues (Gulati, 1993). Completing tasks outside of the households also creates valuable opportunities for social interactions with other women in the community and fosters mutual assistance (Menjívar and Agadjanian, 2007). Such accumulated social capital is beneficial to individual health (Kawachi and Berkman, 2000). Thus, women's increased responsibilities could also bring health benefits.

### 1.3 Women's autonomy as a mechanism

Related to the increased workload and responsibilities, husbands' migration also grants left-behind wives greater decision-making power and autonomy. During the husbands' absence, women in India were more likely to have a say in decisions about what to cook on a daily basis, household expenditures on valuable items, and children's health care and marriage (Desai and Banerji, 2008). In Mozambique, women gained more freedom to go outside to visit friends and relatives, to find a job, to go to the city or district capital, and to receive an HIV test after their husbands have left home (Yabiku et al., 2010). It is also argued that male out-migration promoted secular values and weakened traditional gender norms (Hadi, 2001). The practice of *purdah* appeared to be less common among women married to migrants than among women married to non-migrants in India (Desai and Banerji, 2008). The enhanced autonomy and decision-making power can benefit women's physical health by providing them greater control over their lives and the ability to engage in health-promoting behaviors and seek medical care when needed. For instance, previous studies found that women's decision-making power was positively related to uptaking maternal health care services (Becker et al., 2006; Bloom et al., 2001; Hou and Ma, 2012). Decision-making power and autonomy were found to be associated with lower risks of hypertension among women in India (Stroope, 2015). Therefore, husbands' migration should increase women's autonomy and decision-making power, which are conducive to women's health.

### 1.4 The role of living arrangement

In the patriarchal and patrilocal family system in India, married couples often live with their husband's parents. Living arrangement is an important intervening factor determining how the absence of husbands may change women's lives. On the one hand, extended families can provide companionship and social support to the left-behind wives (De Snyder, 1993; Menjívar and Agadjanian, 2007). Assistance from other adults in the family could compensate for the absence of the husbands' labor, making the wives less overwhelmed by newly assumed responsibilities. Thus, left-behind wives in extended family households may experience less drastic changes in work hours, roles, and responsibilities.

On the other hand, when living with extended families, women are subject to stricter supervision and regulation from senior household members, especially from parents-in-law. Therefore, wives living with extended families after their husbands migrated do not gain as much decision-making power and autonomy as women in nuclear families (Desai and Banerji, 2008; Kaur, 2020). For example, even though the husbands are absent, the women still need to ask for permission from their parents-in-law to go out of the households and consult them for decisions on various household affairs. Another example is that left-behind wives living with extended families still need to eat separately from males when their husbands are absent, while wives in nuclear families are not obligated to do so if no other adult males are present in the household. Furthermore, in extended family households, the male relatives are more likely to receive and manage the remittances sent by the migrants and then allocate part of the money to the left-behind wives, limiting women's control over financial resources. Therefore, we expect that staying-behind wives who live with extended families gain less decision-making power and physical and financial autonomy than left-behind wives in nuclear families. Thus, the potentially positive health impacts of husbands' absence transmitted through improved autonomy are expected to be less prominent among women in extended family households than among those in nuclear families.

## 2 Data and methods

### 2.1 Data

This paper uses data from two waves of the Indian Human Development Survey (IHDS), conducted in 2004–2005 and 2011–2012. While the IHDS-1 interviewed a nationally representative sample of 41,554 households containing 215,751 individuals, the IHDS-2 re-interviewed 83% of the original households as well as the households split from the original households that were residing within the same locality. The sample is spread across 34 states and union territories, and spans 971 urban blocks and 1503 villages in 388 districts of India. The IHDS collected information on household economic activities, social networks, living standards, migration and remittances, and healthcare utilization and expenditure. This survey also asked about demographic characteristics, education, work status, income, and health conditions for each household member. One ever-married woman aged 15–49 years old in each household responded to the eligible women questionnaire in the IHDS-1 and followed up in the IHDS-2, providing information about marriage, fertility, family planning, and gender relations in the household and community. The IHDS-2 followed about 82% of the original sample of eligible women. Due to fewer residential changes in rural areas, the IHDS followed 86% of the rural women as opposed to 70% of the urban ever-married women. This led to slightly more attrition among educated and high-income women.

In the analysis, we focused on 21,245 women aged 15–49 years in both rural and urban India who responded to the eligible women questionnaires in both IHDS-1 and IHDS-2. Then, we excluded 1259 women who were divorced or widowed at the time of the IHDS-2 because they were not subject to the risk of being left behind by a migrant husband. We further dropped 249 cases with missing values on the analytical variables, resulting in a final analytical sample of 19,737 women.

### 2.2 Measures

The dependent variable, women's physical health, was measured using the respondent's rating of their health on a scale ranging from 1 (very poor) to 5 (very good). This question was asked in both the IHDS-1 and IHDS-2. Self-rated health is a commonly used global measure of health status and is a good predictor of mortality in longitudinal studies (Black et al., 2013).

The focal independent variable, the husband's migration status, was based on the husband's place of residence at the time of the IHDS-2 interview. For married women, information was collected about whether their husbands were absent due to out-migration. The binary variable was coded 1 if the husband was absent from the households due to out-migration and coded 0 if the husband was present.

We focus on three sets of mediating variables through which migration may affect women's health: (1) receipt and quantity of remittances; (2) women's responsibilities; and (3) women's autonomy. To capture the economic impact of male out-migration, we measured the remittances received by the left-behind wives during the year before the IHDS-2. A few dummy variables were used to differentiate between wives of non-migrants and wives of out-migrants who received less than 35,000 rupees, between 35,000 and 75,000 rupees, and above 75,000 rupees.

To capture women's responsibilities, we included women's employment status and responsibilities for grocery shopping, animal care, and household financial affairs. Employment status was measured using a categorical variable, distinguishing women who were not working (<240 hours per year), working part-time (240–2000 hours per year), and working full-time (more than 2000 hours per year) on the family farm, in family businesses, and/or in salary jobs. We also included an indicator of whether the woman did food and vegetable shopping for the household. Frequency in animal care was measured by a categorical variable that distinguishes women who never, sometimes, or usually took care of the animals. Responsibility for financial affairs was measured by a binary indicator of whether the woman had any bank account.

Our measures of autonomy included the need for permission to go out, the practice of eating separately, decision-making power, and control over economic resources. We summed the values of three indicators measuring whether a woman needed to ask for permission from her husband or a senior family member to go to a local health center, the home of relatives/friends, or a Kirana shop. The constructed variable had values ranging from 0 (did not need to ask for permission at all) to 3 (needed to ask for permission to go to all three places). A binary variable indicated whether women ate separately from men when the family had the main meal. Women's decision-making power was measured by the total number of household decisions in which they had a say, values ranging from 0 to 6. The household decision-making items included “whether to buy an expensive item,” “how many children to have,” “what to do if a child falls sick,” “what to do if you fall sick,” “whether to buy land or property,” and “to whom the children should marry.” Finally, we included a binary variable indicating whether the woman had cash in hand to spend on household expenditures. The measures of remittances, responsibilities, and autonomy were all taken from the IHDS-2.

We controlled for an array of individual and household characteristics. Woman's age in years and years of completed education by the IHDS-2 were included. We distinguished between women who lived in a nuclear family with no married adult relatives living under the same roof and women who lived with extended families, including the parent(s)-in-law or other married adult relatives in the IHDS-2. Household socioeconomic status was captured by Caste and religious groups, household wealth, land ownership, business ownership, and possession of animals. To capture Caste and religious groups, we used dummy variables to contrast other backward classes (OBC), Dalits, Adivasis, Muslims, and other religious groups (including Christian, Jain, and Sikh) to forward castes. Household assets were originally measured by a sum of 30 items indicating household possessions and housing quality. We then categorized household assets into five quintiles. Household land ownership and business ownership were binary indicators. Household assets, land ownership, and business were measured in the IHDS-1 before the husbands migrated. A dummy variable was included to distinguish urban areas from rural areas. We also measured the total number of children under age 15 in the household and whether the household kept any animals in IHDS-2 to account for the burden of women's workload.

### 2.3 Analytical strategy

We used ordinal logistic regression models with a lagged dependent variable (LDV) to compare the self-rated health of women whose husbands were absent due to migration and women whose husbands were present in the household, controlling for various individual and family characteristics and women's previous health status measured in IHDS-1. Male migration is influenced by a wide variety of factors, some of which may be unobserved and simultaneously affect wives' health. We assumed that unobserved personal and family traits that affect women's health in IHDS-2 also affect their health in IHDS-1. Controlling for the LDV allowed us to capture these unobserved traits that may determine the family's decision to send the male migrant and simultaneously predispose the wives to better or worse health. After we control for the LDV, the remaining variation in the outcome health variable is mainly due to changes that happened between the IHDS-1 and IHDS-2. Thus, although the LDV model is a conservative strategy that reduces the explainable variation in the outcome and increases the standard errors for the coefficient estimates, it facilitates the identification of the causal relationship between husbands' out-migration and wives' health.

Then, we examined several pathways through which husbands' migration influenced wives' health, including changes in women's economic resources, responsibilities, and autonomy. Because living arrangements condition the impact of husbands' absence on women's lives, we examined the mediating mechanisms separately for women in nuclear families and women in extended families. We first added one group of potential mediators at a time into a series of ordered logistic regression models predicting self-rated health. These models showed the effect of mediators on self-rated health and how the coefficient of husbands' migration changed after mediators were added. A comparison of coefficients across logistic regression models is not straightforward because of the rescaling of the model when including new covariates (Breen et al., 2013). Therefore, we conducted a formal test for the indirect effects through each proposed mediator using the procedure developed by Karlson et al. (2012) and implemented through the Stata command *khb* (Kohler et al., 2011). Through the mediation analysis, we separate the positive and negative indirect effects of husbands' out-migration.

## 3 Results

### 3.1 Descriptive analysis of husbands' migration and Women's lives

Table 1 presents the descriptive statistics for our dependent variables and independent variables disaggregated by living arrangement and husbands' migration status. The average self-rated health at the IHDS-2 was 3.9 on a scale from 1 to 5, indicating very good health. For women in nuclear families, wives of migrants rated their health lower than wives of non-migrants (3.81 vs. 3.88), but no significant difference by husbands’ migration status was observed among women living with extended families. In this sample, about 5.3% of women were left behind by migrant husbands. Among all the left-behind wives, about 42% received remittances less than 35,000 rupees (2.2% in the total sample), 32% received 35,000 to 75,000 rupees (1.7% in the total sample), and 28% received more than 75,000 rupees (1.5% in the total sample) in the past year.

**Table 1:** Descriptive statistics of variables in the analysis of husbands' our-migration and women's health in India, IHDS-1 and IHDS-2.

		Nuclear family		Extended family	
	Full sample	Non-migrant	Migrant	Non-migrant	Migrant
**Dependent variable**					
Self-rated health	3.90	3.88	3.81*	3.94	3.87
**Mediators**					
*Measure of economic resources*					
Remittances					
Non-migrant	.947	.942	–	.954	–
Less than 35,000 rupees	.022	–	.024	–	.018
35,000–75,000 rupees	.017	–	.019	–	.012
75,000 rupees and above	.015	–	.015	–	.016
*Measures of responsibilities*					
Employment status					
Not employed	.44	.44	.43	.44	.47
Part-time employed	.47	.46	.49	.47	.48
Full-time employed	.09	.09	.08	.09	.06
Grocery shopping	.63	.65	.81***	.58	.50**
Frequency of animal care					
No	.61	.63	.52***	.59	.50**
Sometimes	.10	.08	.04	.13	.18
Usually	.29	.28	.44	.29	.32
Bank account holder	.41	.41	.56***	.39	.49***
*Measures of autonomy*					
Needs permission to go out	2.17	2.16	1.58***	2.25	2.16
Women eat separately	.40	.36	.32+	.48	.55**
Decision-making power	5.09	5.19	5.46***	4.92	4.70*
Cash to spend	.93	.94	.99***	.92	.92
**Control variables**					
Self-rated health in wave 1	3.78	3.77	3.69**	3.81	3.84
Age	37.68	37.98	36.91***	37.37	35.78***
Years of ducation	4.6	4.36	4.34	4.99	5.26
Caste and religion groups					
Forward castes	.22	.20	.24***	.24	.23***
Other backward classes	.34	.34	.31	.34	.40
Dalit	.22	.23	.20	.20	.17
Adivasi	.08	.09	.04	.08	.03
Muslim	.12	.12	.18	.10	.14
Christian, Sikh, Jain	.03	.02	.03	.03	.03
Household assets in wave 1					
First quintile	.16	.19	.24**	.12	.15
Second quintile	.18	.18	.18	.15	.17
Third quintile	.23	.25	.20	.25	.26
Fourth quintile	.23	.20	.18	.23	.17
Fifth quintile	.20	.18	.19	.25	.25
Business ownership in wave 1	.23	.22	.12***	.24	.17***
Land ownership in wave 1	.46	.40	.55***	.54	.72***
Animal ownership	.47	.41	.50***	.55	.68***
Urban residence	.30	.33	.18***	.28	.13***
Number of children	1.66	1.45	1.87***	1.92	2.58***
Number of observations	19,737	11,489	703	7201	344

Notes: ***p < 0.001, **p < 0.01, *p < 0.05, + p < 0.1.

Asterisks are based on t-tests or Chi-square tests comparing the characteristics of wives of non-migrants and wives of migrants.

Reflecting the low female labor force participation in India, 44% of women in this sample were not employed, 47% were employed part-time, and only 9% were employed full-time. The pattern of employment status did not differ by the husbands’ migration status for women under either living arrangement. About 63% of the women did grocery shopping for their families. The wives living in nuclear households were significantly more likely to do so if the husbands were absent. However, when living in extended families, left-behind wives were less likely than wives of non-migrants to do grocery shopping. In general, about 61% of women did not take care of animals, 10% of women took care of animals sometimes, and 29% did it usually. In nuclear families, a much higher percentage (44%) of women took full responsibility for animal care when their husbands migrated. In extended families, some left-behind wives took partial responsibility for animal care (18%), and some took full responsibility when their husbands are absent (32%) because they receive help from extended family members. In terms of responsibility for managing financial affairs, left-behind women were significantly more likely to hold a bank account than wives of non-migrants in both living arrangements.

Regarding the measure of autonomy, on average, women needed to ask for permission to go to two out of three places listed in the survey. In nuclear families, women were less likely to be required to ask for permission if their husbands were away, but this was not the case for women in extended families. Two-fifths of women had to eat separately from men when having main meals. The wives in nuclear families were less likely to do so when their husbands migrated. However, women in extended families became more likely to eat separately when their husbands were away. This pattern is consistent with the norm of purdah, which requires women's physical segregation from male relatives. (Note that a substantial number of women in nuclear families still said that they need to ask for permission to go out and eat separately even when their husbands were absent. Thus, their responses may reflect the perceived norms in the households rather than the actual behaviors.) On average, women in this sample had a say in five out of six family decisions. Women living in nuclear families gained greater decision-making power after husbands' out-migration. In contrast, women in extended families became less involved in decision-making after their husbands migrated. Regarding economic autonomy, 93% of women had cash to spend. For women living in nuclear households, out-migration of the husband was associated with a higher likelihood of owning cash. For women living in extended families, left-behind wives were not more likely than wives of non-migrants to have cash, reflecting their restricted autonomy when living with elderly relatives.

For control variables, the mean self-rated health was 3.78 at the IHDS-1. The average age of the sampled women was 37.78. The average years of completed education was only 4.6 years. About one-fifth of the families belonged to the forward Castes, one-third belonged to Other Backward Classes, one-fifth were Dalits, and the remaining were Adivasis, Muslims, or other religious groups. This sample included more families located at higher asset quintiles than families located at lower asset quintiles. About 46% of the families owned land, 22% of families owned business, about half owned animals, and 30% lived in urban areas. The households, on average, contained 1.73 children under age 15. It is worth noting that left-behind wives tend to be younger, less wealthy, less likely to own business, and more likely to own land and animals than wives of non-migrants. They also lived in households with more children, probably because of their rural residence and lower socioeconomic status.

### 3.2 Total effect of husbands' migration on Women's health

Table 2 presents the ordered logistic regression models with LDV predicting women's self-rated health in the IHDS-2. The coefficient (b = −0.173) indicated that left-behind wives rated their health lower than wives of non-migrants after accounting for various individual and family characteristics and their previous health status. Although husbands' out-migration could influence women's health through both positive and negative pathways, this negative total effect suggests that the detrimental health influence of husbands' absence cannot be fully compensated by the potential positive mechanisms.

**Table 2:** Lagged dependent variable models assessing the main effect of husbands’ out-migration on the health of wives in India.

	Ordered Logistic Regression
Husband's migration status	
Non-migrant (ref.)	
Husband is migrant	−0.173**
Self-rated health in wave 1	0.115***
Extended family	0.080**
Age	−0.017***
Years of education	0.026***
Caste and religion groups	
Forward castes (ref.)	
Other backward classes	0.106**
Dalit	0.001
Adivasi	0.304***
Muslim	−0.210***
Christian, Sikh, Jain	0.417***
Household assets in wave 1	
First quintile (ref.)	
Second quintile	0.105*
Third quintile	0.127**
Fourth quintile	0.243***
Fifth quintile	0.429***
Business ownership in wave 1	−0.086*
Land ownership in wave 1	−0.067*
Animal ownership	−0.166***
Urban residence	−0.059
Number of children	0.021*
Constant cut1	−5.597***
Constant cut2	−2.436***
Constant cut3	−1.130***
Constant cut4	1.310***
	
Observations	19,737
Log Likelihood	−23,015

Notes: ***p < 0.001, **p < 0.01, *p < 0.05, + p < 0.1.

The effects of most control variables in this model were in expected directions. Self-rated health in the IHDS-1 was positively associated with women's self-rated health in the IHDS-2. Women who were younger, with more years of education, and living in extended families had better self-rated health than their counterparts. Compared with women who belonged to forward Castes, women in other backward classes, Adivasis, Christians, Jains, and Sikhs rated their health higher. Muslim women rated their health lower than women in the forward Castes. More family assets were associated with better self-rated health. However, owning family businesses and animals had a negative relationship with self-rated health, which might be attributable to the burden of taking care of family businesses and animals.

### 3.3 Robustness checks for the total effect

We check the robustness of the migration effect using the propensity score approach and fixed-effect models. In the propensity score analysis, we first estimated a logistic regression to predict the propensity of having a migrant husband in the IHDS-2 given individual, household, and community characteristics measured in the IHDS-1 (Appendix A). Predicted values from this logistic regression are used to estimate the propensity score. We selected one case in the control group for each treated case using the nearest propensity score within the caliper of 0.25 standard deviation of the logged propensity score. All but one case fell into the common support. Based on results from t-tests and Chi-square tests (Appendix B), balance had been achieved for almost all the matching variables. Then, an ordered logistic regression was estimated using the matched sample (1,014 control cases and 1,014 treated cases) to compute the treatment effect (column 1 in Appendix C). We also estimated ordered logistic regressions using the full sample (N = 19,686) weighted by the inverse of the propensity score, without covariates and with covariates (columns 2 and 3 in Appendix C). Results from all three methods show that if a woman were left behind by a migrant husband, she would be 13%–20% less likely to provide a higher rating on health. The magnitude of the effect is comparable to the effect reported in the LDV model in Table 2.

Next, we measured husbands' migration status from both IHDS-1 and IHDS-2. We excluded women whose husbands were migrants in wave 1 but returned by wave 2 and only focused on women whose husbands were present at home in wave 1. The fixed-effect model (column 4 in Appendix C) compares the changes in the health of women whose husbands became migrants by wave 2 with the changes in the health of women whose husbands were non-migrants in both waves. The result shows that out-migration reduces women's health, but the coefficient was marginally significant (*p* = 0.057).

### 3.4 Indirect effects of husbands' migration through changes in Women's lives

Next, we examined whether women's economic resources, responsibilities, and autonomy explain the relationship between husbands' out-migration and women's health, separately for women in nuclear families and women in extended families. We separately analyze women in nuclear families and women in extended families because they face different restrictions and power dynamics within the households. Descriptive statistics in Table 1 showed that the impact of husbands' absence on women's lives vary by living arrangements, which led us to expect different mechanisms through which husbands' migration influences the health of women in different types of households. Table 3 presents models for women living in nuclear families in IHDS-2. Model 1 is the baseline model showing the total effect of husbands' migration status on women's self-rated health. The negative and significant coefficient (b = −0.169) indicates that left-behind wives rated their health lower than wives of non-migrants. In Model 2, we assessed the role of remittances by categorizing left-behind wives into three groups. Left-behind wives who received remittances less than 35,000 rupees in the previous year rated their health significantly lower than wives of non-migrants, while left-behind women who received more than 35,000 rupees did not report worse health than women married to a non-migrant. Based on these results, the detrimental health effect of husbands' out-migration was driven by the effect among women who receive no or little remittances. (However, in our sensitivity analysis, we did not find a significant difference between left-behind women who received less than 35,000 rupees and those who received more than 35,000 probability due to the small number of cases in each category.)

**Table 3:** Ordered logistic regression models predicting the self-rated health of women in nuclear families.

	Baseline model	Model with remittances	Model with responsibilities	Model with autonomy
	(1)	(2)	(3)	(4)
Husband's migration status				
Non-migrant (ref.)				
Husband is migrant	−0.169*	–	−0.169*	−0.215**
*Measure of economic resources*				
Remittances				
Non-migrant (ref.)				
Less than 35,000 rupees		−0.262*		
35,000–75,000 rupees		−0.090		
Above 75,000		−0.124		
*Measures of responsibilities*				
Employment status				
Not employed (ref.)				
Part-time employed			−0.022	
Full-time employed			0.107+	
Grocery shopping			0.190***	
Frequency of animal care				
No				
Sometimes			0.042	
Usually			−0.248**	
Bank account holder			−0.102**	
*Measures of autonomy*				
Needs permission to go out				0.022
Women eat separately				−0.264***
Decision-making				0.054***
Cash to spend				0.511***
				
Observations	12,192	12,192	12,192	12,192
Log-likelihood	−14364	−14364	−14335	−14301

Note: a. ***p < 0.001, **p < 0.01, *p < 0.05, + p < 0.1.

b. The models in this table controlled for women's age, education, self-rated health at IHDS-1, Castes and religious groups, household assets, land ownership, business ownership, animal ownership, urban residence, and the number of children in the households.

In Model 3, we added measures of women's responsibilities to the baseline model. Compared with women who were not employed, those who were employed full-time had better self-rated health. Women who were responsible for grocery shopping also reported better health, possibly because they gained freedom of physical mobility and control over household expenditures through this responsibility. However, women who usually took care of animals had worse health than women who did not take care of animals. Besides, holding a bank account was negatively associated with women's health. After these measures were included, the coefficient of the husband's migration barely changed, probably because these variables capture both positive and negative mechanisms. We specifically test the mechanism through each variable below.

Model 4 in Table 3 added measures of women's autonomy to the baseline model. Although the need for permission to go out did not affect self-rated health, eating separately from men was associated with poorer self-rated health. Decision-making power and having cash to spend were associated with better self-rated health. After including these variables, the negative effect of husbands' migration became even stronger. Because the absence of husbands provides wives more power and autonomy within the households and the improved status of woman, in turn, has positive effects on health, the negative health impact of husbands' migration would have been larger if there were no positive changes in women's autonomy.

As stated above, it is not straightforward to interpret the changes in coefficients across ordered logistic regressions due to the rescaling issue. Table 4 presented the results of formal mediation analysis taking the rescaling bias into account. According to the results, two of the mediators explained a significant proportion of the negative impact of the husband's out-migration on the health of women in nuclear families. Women's increased responsibility for animal care explained 13.7% of the negative impact (Column 3, Table 4), and holding a bank account explained 8.8% of the negative health implications (Column 4). Taken together, these two mechanisms explained 22.6% of the negative effect of husbands' absence due to migration (column 9).

**Table 4:** Mediation analyses for the relationship between the Husband's out-migration and the health of women in nuclear families.

	Measures of responsibility				Measures of autonomy				Total positive and negative indirect effects	
	Employment status	Grocery shopping	Animal care	Bank account holder	Needs permission	Eats separately	Decision-making	Cash to spend	Animal care + Bank account holder	Grocery + Eats separately + Decision making + Cash
	(1)	(2)	(3)	(4)	(5)	(6)	(7)	(8)	(9)	(10)
Total effect of migration	-.170*	-.170*	-.168*	-.170*	-.169*	-.171*	-.169*	-.169*	-.168*	-.170*
Direct effect of migration	-.170*	-.207**	-.144+	-.155*	-.156*	-.184*	-.186*	-.201*	-.131	-.248**
Indirect effect through mediators	-.000	.037***	-.023***	-.015*	-.013	.013*	.017***	.032***	-.038***	.078***
Percentage explained	0%	−21.7%	13.7%	8.8%	7.7%	−7.6%	−10.1%	−18.9%	22.6%	−45.9%

Note: N = 12,192, ***p < 0.001, **p < 0.01, *p < 0.05, + p < 0.1.

The mediation analysis in Table 4 also showed the positive pathways through which husbands' out-migration benefited the health of women in nuclear families. Gaining opportunities to do grocery shopping, eating together with other family members, making more decisions, and having cash to spend all brought positive health implications for women whose husbands migrated. Each of the factors suppressed between 7% and 21% of the total effect of husbands' out-migration. If it had not been for these positive changes in women's autonomy and decision-making power, the total negative impact of husbands' out-migration would have been 46% stronger (Column 10).

Parallel analyses of mediating effects were carried out for women in extended families. Results from ordered logistic regressions were presented in Table 5. The coefficient of husbands' migration in Model 1 showed that, in extended families, left-behind wives rated their health lower than wives of non-migrants, though the effect was only marginally significant. Coefficients in Model 2 indicated that left-behind wives receiving fewer remittances had poorer self-rated health than wives of non-migrants. However, women who received more than 35,000 rupees were not different from wives of non-migrants in their self-rated health. (Due to small sample sizes in each remittance category, we did not detect significant differences among the health of women who received different levels of remittances.) In Model 3, employment status had no significant relationship with health, but taking care of animals was again associated with worse self-rated health. Similar to women in nuclear families, doing grocery shopping was positively related to self-rated health. Holding a bank account had a negative influence on women's self-rated health. Model 4 included measures of autonomy. Consistent with the results among women in nuclear families, eating separately from males was associated with poorer self-rated health, whereas making more decisions and having cash to spend predicted better self-rated health.

**Table 5:** Ordered logistic regression models predicting the self-rated health of women in extended families.

	Baseline model	Model with remittances	Model with responsibilities	Model with autonomy
	(1)	(2)	(3)	(4)
Husband's migration status				
Non-migrant (ref.)				
Husband is migrant	−0.174+	–	−0.154	−0.165
*Measure of economic resources*				
Remittances				
Non-migrant (ref.)				
Less than 35,000 rupees		−0.296+		
35,000–75,000 rupees		−0.081		
Above 75,000		−0.113		
*Measures of responsibilities*				
Employment status				
Not employed (ref.)				
Part-time employed			−0.076	
Full-time employed			0.045	
Grocery shopping			0.184***	
Frequency of animal care				
No				
Sometimes			−0.136	
Usually			−0.230**	
Bank account holder			−0.153**	
*Measures of autonomy*				
Needs permission to go out				0.012
Women eat separately				−0.286***
Decision-making				0.028*
Cash to spend				0.439***
				
Observations	7545	7545	7545	7545
Log-likelihood	−8640	−8640	−8621	−8600

Note: a. ***p < 0.001, **p < 0.01, *p < 0.05, + p < 0.1.

b. The models in this table controlled for women's age, education, self-rated health at IHDS-1, castes and religious groups, household assets, land ownership, business ownership, animal ownership, urban residence, and the number of children in the households.

The KHB analyses presented in Table 6 examined the indirect effect through each of the potential mediators. Like women in nuclear families, about 11% of the negative health impact of husbands' absence was transmitted through the added responsibility of holding a bank account (Column 4). For women living in extended-family households, left-behind wives were more likely than wives of non-migrants to eat separately from men, which was detrimental to their health. The increased likelihood of having separate meals accounted for 8.1% of the total negative effect of husbands' out-migration on women's health (Column 6). Eating separately from male relatives and holding a bank account together explained one-fifth of the effect of husbands' out-migration on the health of women who resided with extended relatives.

**Table 6:** Mediation analyses for the relationship between the Husband's out-migration and the health of women in extended families.

	Measures of responsibility				Measures of autonomy				Total indirect effect
	Employment status	Grocery shopping	Animal care	Bank account holder	Needs permission	Eats separately	Decision-making	Cash to spend	Eats separately + Bank account holder
	(1)	(2)	(3)	(4)	(5)	(6)	(7)	(8)	(9)
Total effect of migration	-.174+	-.175+	-.173	-.175+	-.174+	-.172	-.176	-.175+	-.173
Direct effect of migration	-.176+	-.171	-.174+	-.155	-.173	-.158	-.172	-.184+	-.138
Indirect effect through mediators	.002	-.004	.001	-.019**	-.001	-.014+	-.004	.009	-.035**
Percentage explained	1.1%	2.3%	0.5%	10.9%	0.6%	8.1%	2.3%	5.1%	20.2%

Note: N = 7,545, ***p < 0.001, **p < 0.01, *p < 0.05, + p < 0.1.

## 4 Discussion and conclusion

In this study, we employed data from the IHDS to examine the relationship between husbands' out-migration and the health of the wives in India, a country with ingrained gender inequality. The findings from the analyses allowed us to draw several conclusions. First, the results from the LDV models, propensity score analyses, and fixed-effect models indicated that the absence of husbands due to migration had a negative overall effect on wives' self-rated health. This effect held regardless of whether the left-behind wives lived in nuclear families or extended families. Although husbands' out-migration can influence women's health through both positive and negative pathways, it seems that the detrimental health effects cannot be fully offset by the beneficial effects.

We subsequently investigated the mechanisms through which husbands' migration exerts an impact on women's health separately for women with different living arrangements. Remittances played an important role in shaping women's health outcomes for both women in nuclear families and extended families. According to the results, the negative health implications of husbands' out-migration were partly driven by low remittances sent by the migrants, which could cause economic hardship for women left behind and lead to negative health consequences.

We also found evidence that the added responsibilities were detrimental to women's health, which partially explained the negative relationship between husbands' migration and wives' self-rated health. For women in nuclear families, the increased obligation of animal care and the need to hold a bank account together explained 23% of the negative effect of husbands' out-migration. Taking care of animals can increase the physical strain and stress level of women staying behind. Holding a bank account could be difficult for women in Indian society especially given the norms of female seclusion. In extended family households, the responsibility of holding a bank account explained 11% of the negative health impact of male out-migration for women in extended families. Our findings are consistent with a recent study in India, reporting women's increased responsibilities in agricultural work, water fetching, and child care after husbands' migration and a negative consequence for women's food security, a risk factor for poor health (Choithani, 2020). Another study in China also found a detrimental effect of husbands' migration on women's mental health through women's added responsibility of being the household master (Tong et al., 2019).

As expected, we found that left-behind wives in nuclear families gained greater autonomy than left-behind wives in extended families, as the behaviors of the latter groups were more closely monitored and constrained by senior family members. For instance, women in nuclear families were more likely to make decisions in households and have cash to spend when their husbands migrated. They were also less required to ask for permission to go out and to eat separately from male family members after the husbands migrated. These changes suppressed what would be a 46% stronger negative impact of husbands' out-migration on the self-rated health of women in nuclear families. However, no such positive changes in autonomy were observed among women living with extended families. These findings are consistent with previous studies, which reported little gain or even loss of autonomy for women in extended families after husbands’ migration (Desai and Banerji, 2008; Kaur, 2020).

Different from previous studies, our analyses also revealed that women in extended families received support from family members. According to our findings, left-behind wives in nuclear families suffered worse health from the added burden of animal care. In contrast, left-behind wives in extended families did not experience the health toll from the burden of caring for animals, because extended family members shared the responsibility with them.

Admittedly, the unique social context of India could shape our findings and affect their generalizability to other contexts. Due to the unequal gender relationships and female seclusion, husbands' absence may have a more negative impact on women's health in India than in some other countries with more egalitarian gender norms. On the other hand, we expect the indirect impact through remittances to be similar across social contexts, conditioned on whether the remittances are large enough to improve the living standards. The positive pathway through enhanced decision-making power and autonomy should also be generalizable to other contexts with unequal gender relationships, as changes in women's autonomy due to husbands' absence have been reported in other settings (Hadi, 2001; Yabiku et al., 2010).

Moreover, future research will profit from addressing some limitations of this research. First, even though we conducted robustness checks using the propensity score method and fixed-effect model, we are not fully confident to make causal inference due to the potential influence of unobserved time-varying confounders. Second, besides the mechanisms we found, there is still unexplained negative effect of husbands' migration. We suspect that the remaining negative effect of male out-migration may be attributable to the reduced social support and emotional strain related to the spousal absence, for which we, unfortunately, do not have measures. Third, the association between grocery shopping and better health can also be explained by the possibility that healthier women were more likely to be responsible for grocery shopping. Also, we reported a negative effect of holding a bank account on women's health, but qualitative studies are required to reveal how managing a bank account leads to negative health consequences for Indian women.

Our research is based on data collected in 2004–5 and 2011–12, almost a decade ago. Over the past decade, Indian society has experienced several changes in gender relations, some positive and others negative. Notable changes include increasing education and increasing bank account ownership among women between 2005 and 2015 (International Institute for Population Sciences (IIPS) and ICF, 2017; International Institute for Population Sciences (IIPS) and Macro International, 2007). Gender norms, however, were slower to change, and female labor force participation has dropped over time. An interesting avenue for research would be to examine how the impact of male migration on left-behind wives' autonomy and well-being vary by women's education and employment status.

## Credit author statement

Lei Lei: Conceptualization, Methodology, Formal analysis, Writing – original draft, Funding acquisition. Sonalde Desai: Conceptualization, Methodology, Writing – review & editing

## Figures and Tables

**Table:** 

	(1)
Wife's age	0.022+
Wife's education	−0.018+
Husband's age	−0.043***
Husband's education	0.073***
Caste and religion groups	
Forward castes (ref.)	
Other backward classes	−0.060
Dalit	−0.163
Adivasi	−1.042***
Muslim	0.537***
Christian, Sikh, Jain	0.042
Household structure in wave 1	
Only married women (ref.)	
Senior married women	0.538*
Junior married women	0.345***
Household assets in wave 1	
First quintile (ref.)	
Second quintile	−0.088
Third quintile	−0.209+
Fourth quintile	−0.064
Fifth quintile	0.056
Land ownership in wave 1	0.115
Business ownership in wave 1	−0.675***
Animal ownership in wave 1	0.106
Number of children in wave 1	0.077**
Wife's self-rated health in wave 1	−0.061
Community type	
Metropolitan urban (ref.)	
Other urban	1.899***
More developed rural	2.490***
Less developed rural	2.573***
Constant	−4.647***
	
Observations	19,686
Log-likelihood	−3748
Pseudo R square	0.0624

Note: ***p < 0.001, **p < 0.01, *p < 0.05, + p < 0.1.

**Table:** 

	Mean or proportion		
	Non-migrant	Migrant	P value from *t*-test or Chi-square test
Wife's age	36.57	36.51	.402
Wife's education	4.80	4.61	.175
Husband's age	41.19	41.22	.544
Husband's education	7.54	7.58	.594
Caste and religion groups			
Forward castes	.24	.23	.923
Other backward classes	.33	.34	
Dalit	.21	.20	
Adivasi	.03	.03	
Muslim	.16	.16	
Christian, Sikh, Jain	.03	.03	
Household structure in wave 1			
Only married women	.57	.58	.480
Senior married women	.02	.02	
Junior married women	.42	.10	
Household assets in wave 1			
First quintile	.16	.21	.004
Second quintile	.16	.18	
Third quintile	.28	.22	
Fourth quintile	.19	.17	
Fifth quintile	.19	.21	
Land ownership in wave 1	.62	.61	.411
Business ownership in wave 1	.14	.14	.701
Animal ownership in wave 1	.58	.58	1.000
Number of children in wave 1	2.28	2.26	.439
Wife's self-rated health in wave 1	3.75	3.74	.467
Community type			
Metropolitan urban	.01	.01	.988
Other urban	.15	.15	
More developed rural	.38	.39	
Less developed rural	.46	.46	
Observations	1014	1014	

**Table:** 

	Propensity score matching	Inverse probability weighting	Inverse-probability -weighted regression adjustment^a^	Fixed-effect ordered logistic regression
	(1)	(2)	(3)	(4)
Average Treatment Effect of husbands' out-migration	-.140+	-.220**	-.212*	-.216+
	(.084)	(.072)	(.072)	(.113)
				
Number of observations	2028	19,686	19,686	36,160

Notes: ***p < 0.001, **p < 0.01, *p < 0.05, standard errors in parentheses.

a This regression includes all covariates in Table 2.
